# Iron, Anemia, and Iron Deficiency Anemia among Young Children in the United States

**DOI:** 10.3390/nu8060330

**Published:** 2016-05-30

**Authors:** Priya M. Gupta, Cria G. Perrine, Zuguo Mei, Kelley S. Scanlon

**Affiliations:** Division of Nutrition, Physical Activity, and Obesity, Centers for Disease Control and Prevention, Atlanta, GA 30341, USA; hgk3@cdc.gov (C.G.P.); zam0@cdc.gov (Z.M.); kxs5@cdc.gov (K.S.S.)

**Keywords:** iron deficiency, anemia, NHANES

## Abstract

Iron deficiency and anemia are associated with impaired neurocognitive development and immune function in young children. Total body iron, calculated from serum ferritin and soluble transferrin receptor concentrations, and hemoglobin allow for monitoring of the iron and anemia status of children in the United States. The purpose of this analysis is to describe the prevalence of iron deficiency (ID), anemia, and iron deficiency anemia (IDA) among children 1–5 years using data from the 2007–2010 National Health and Nutrition Examination Survey (NHANES). Prevalence of ID, anemia, and IDA among children 1–5 years was 7.1% (5.5, 8.7), 3.2% (2.0, 4.3), and 1.1% (0.6, 1.7), respectively. The prevalence of both ID and anemia were higher among children 1–2 years (*p* < 0.05). In addition, 50% of anemic children 1–2 years were iron deficient. This analysis provides an update on the prevalence of ID, anemia, and IDA for a representative sample of US children. Our results suggest little change in these indicators over the past decade. Monitoring of ID and anemia is critical and prevention of ID in early childhood should remain a public health priority.

## 1. Introduction

Iron deficiency (ID) is the most common nutritional deficiency in the world and infants and young children are at the highest risk [1]. Iron deficiency in young children significantly increases the risk of developmental delays and behavioral disturbances. It is also known to cause iron deficiency anemia (IDA) [2].

The objective for this analysis is to provide an update on the prevalence of ID, anemia, and IDA among children 1–5 years in the United States.

## 2. Materials and Methods

We analyzed data from the 2007–2010 National Health and Nutrition Examination Survey (NHANES), a cross-sectional survey representative of the total non-institutionalized civilian population in the United States. NHANES uses a stratified multistage, probability design to select participants and is conducted via household interviews and standardized examinations in the NHANES mobile examination centers [3,4].

Children 1–5 years (12–71.9 months) who had complete nutritional biochemistry data on serum transferrin receptor (sTfR), ferritin, and hemoglobin were included in our analysis (*n* = 1156). Starting in 2004, serum ferritin and sTfR concentrations were measured by the RocheTina-quant immunoturbidimetric assay on the Hitachi 912 clinical analyzer Roche Diagnostics. Hemoglobin was measured as part of a complete blood count done on the Coulter^®^ HMW [5,6,7]. Total body iron (TBI) is the suggested indicator for ID in the United States [8]. We calculated TBI on the basis of sTfR and ferritin concentrations through Equation (1) [5,9]:

TBI (mg/kg) = −[log10 (sTfR × 1000/ferritin) − 2.8229]/0.1207(1)

For this calculation we converted Roche sTfR concentrations to those equivalent to the Flowers assay used in the development of the body iron model [5,10], see Equation (2):

Flowers sTfR = 1.5 × Roche sTfR + 0.35 mg/L(2)

ID was defined as TBI < 0 mg/kg. Anemia was defined as hemoglobin concentration <11.0 g/dL [11,12]. IDA was defined as having both anemia and ID. Estimates were weighted and take into account NHANES complex survey design. We used chi square tests to assess whether prevalence estimates varied by age.

## 3. Results

Among children aged 1–5 years, median total body iron was 4.0 mg/kg (95% CI: 3.9, 4.2) and mean hemoglobin was 12.6 g/dL (95% CI: 12.5, 12.7). Among children aged 1–5 years, 7.1% were iron deficient. The prevalence of anemia and IDA were 3.2% and 1.1%, respectively (Table 1) Approximately 35% of children who were anemic were iron deficient.

The prevalence of both ID and anemia were higher among children aged 1–2 years (*p* < 0.05). Among younger children, 5.4% were anemic and 2.7% had IDA. Therefore, approximately 50% of children 1–2 years who were anemic were iron deficient.

## 4. Discussion

This analysis provides an update on the iron and anemia status of children 1–5 years in the United States. Previous analysis of NHANES 2003–2006 data showed that 14.4% of children 1–2 years and 3.7% of children 3–5 years were iron deficient [14]. Our results are similar and confirm that ID among young children in the United States remains a public health concern. Extrapolation of the prevalence estimate of ID to U.S. census data suggests that approximately 1.5 million children between the ages of 1 and 5 years may be at risk for iron deficiency. Although the definition of anemia differed slightly for children 24–59 months (≤111 g/L *vs.* ≤110 g/L), an analysis, of children 12–59 months (1–4 years), using NHANES 1999–2002 data found that 3.6% were anemic and the prevalence of IDA was 1.2% (Iron deficiency anemia was defined as “anemia plus abnormal value ≥2: serum ferritin, transferrin saturation, and erythrocyte protoporphyrin”) [11]. Our results showing the prevalence of anemia and IDA, among children 1–5 years, as 3.2% and 1.1% respectively, suggests little change in these indicators over the past decade. Iron deficiency and anemia are associated with impaired psychomotor and neurocognitive development as well as impaired immune function in children and often these consequences are irreversible [15,16,17,18]. Therefore, prevention of ID in early childhood should remain a public health priority.

## Figures and Tables

**Table 1:** Prevalence of Iron Deficiency, Anemia, and Iron Deficiency Anemia among Children 1–5 years in the United States, National Health and Nutrition Examination Survey (NHANES) 2007–2010.

	*n*	Iron Deficiency ^1^		Anemia ^2^		Iron Deficiency Anemia ^3^	
		%	95% Confidence Interval	%	95% Confidence Interval	%	95% Confidence Interval
1–5 years (12–71.9 months)	1437	7.1	(5.5, 8.7)	3.2	(2.0, 4.3)	1.1	(0.6, 1.7)
1–2 years (12–35.9 months)	643	13.5 *	(9.8, 17.2)	5.4 *	(3.5, 7.4)	2.7	(1.2, 4.2)
3–5 years (36–71.9 months)	794	3.7	(1.9, 5.5)	1.9	(0.7, 3.1)	- **	-

^1^ Iron deficiency was defined as total body iron (TBI) <0 mg/kg; ^2^ Anemia was defined as hemoglobin concentration <11.0 g/dL; ^3^ Iron deficiency anemia was defined as having both anemia and iron deficiency; * Chi. Square test *p*-value < 0.05 for differences between children 1–2 years and 3–5 years; ** The Centers for Disease Control and Prevention’s National Center for Health Statistics does not support presenting estimates when relative standard errors (standard error of prevalence/prevalence) are ≥30% so this estimate was suppressed [13].

## Abbreviations

The following abbreviations are used in this manuscript:

ID	Iron deficiency
IDA	Iron deficiency anemia
sTfR	Serum transferrin receptor
