# Correction: Gupta, P.M.; et al. Iron, Anemia, and Iron Deficiency Anemia among Young Children in the United States *Nutrients* 2016, *8*, 330

**DOI:** 10.3390/nu9080876

**Published:** 2017-08-15

**Authors:** Priya M. Gupta, Cria G. Perrine, Zuguo Mei, Kelley S. Scanlon

**Affiliations:** Division of Nutrition, Physical Activity, and Obesity, Centers for Disease Control and Prevention, Atlanta, GA 30341, USA; hgk3@cdc.gov (C.G.P.); zam0@cdc.gov (Z.M.); kxs5@cdc.gov (K.S.S.)

We would like to submit the following correction to our recently published paper [1] due to the error in classification of children as anemic. The details are as follows:

(1) The sentence in abstract “Prevalence of ID, anemia, and IDA among children 1–5 years was 7.1% (5.5, 8.7), 3.2% (2.0, 4.3), and 1.1% (0.6, 1.7), respectively.” has been changed as “Prevalence of ID, anemia, and IDA among children 1–5 years was 7.1% (5.5, 8.7), 3.9% (2.0, 4.3), and 1.1% (0.6, 1.7), respectively.”
(2) In the last paragraph of Section 2, the sentence “Anemia was defined as hemoglobin concentration <11.0 g/dL” has been changed as “Anemia was defined as hemoglobin concentration <11.0 g/dL for children 6–59 months and <11.5 g/dL for children 60–72 months”.
(3) In Section 3, the last two sentences have been changed as “The prevalence of anemia and IDA were 3.9% and 1.1%, respectively (Table 1). Approximately 28% of children who were anemic were iron deficient”.
(4) Table 1 has been changed as:
(5) The first sentence of the last paragraph in Section 3 has been changed as “The prevalence of ID was higher among children aged 1–2 years (*p* < 0.05)”.
(6) In Section 4, the sentence “Our results showing the prevalence of anemia and IDA, among children 1–5 years, as 3.2% and 1.1% respectively, suggests little change in these indicators over the past decade.” has been changed as “Our results showing the prevalence of anemia and IDA among children 1–5 years as 3.9% and 1.1%, respectively, suggests little change in these indicators over the past decade.”

We apologize for any inconvenience caused to our readers.

## Figures and Tables

**Table 1:** Prevalence of iron deficiency, anemia, and iron deficiency anemia among children 1–5 years in the United States, National Health and Nutrition Examination Survey (NHANES) 2007–2010.

	*n*	Iron Deficiency ^1^		Anemia ^2^		Iron Deficiency Anemia ^3^	
		%	95% Confidence Interval	%	95% Confidence Interval	%	95% Confidence Interval
1–5 years (12–71.9 months)	1437	7.1	(5.5, 8.7)	3.9	(2.5, 5.3)	1.1	(0.6, 1.7)
1–2 years (12–35.9 months)	643	13.5 *	(9.8, 17.2)	5.4	(3.5, 7.4)	2.7	(1.2, 4.2)
3–5 years (36–71.9 months)	794	3.7	(1.9, 5.5)	3.1	(1.4, 4.7)	- **	-
